# Medical monitoring of asbestos-exposed workers: experience from Poland

**DOI:** 10.2471/BLT.15.159426

**Published:** 2016-06-02

**Authors:** Beata Świątkowska, Neonila Szeszenia-Dąbrowska, Urszula Wilczyńska

**Affiliations:** a Department of Occupational and Environmental Epidemiology, Reference Center for Asbestos Exposure & Health Risk Assessment, Nofer Institute of Occupational Medicine, Sw. Teresy 8, 91–348 Lodz, Poland.

## Abstract

In Poland, the use of asbestos was banned in 1997 and asbestos plants have been closed since then. Despite their closure, cases of asbestos-related occupational diseases among former asbestos workers are still being recorded in the Central Register of Occupational Diseases. Between 2001 and 2014, there were 2726 asbestos-related illnesses, classified and reported as diseases associated with occupational exposure to asbestos. In 2000, Poland introduced a programme called *Amiantus*, targeted at former asbestos-processing plant workers. The programme provided periodic medical examinations to workers and free access to medications for treatment of asbestos-related illnesses. Introduction of the programme provided additional data to generate a reliable estimation of the number of asbestos-related occupational diseases, including cancer. The average latency period for asbestosis, lung cancer and mesothelioma is about 40 years so there may still be some health impact to former workers necessitating follow-up. We present the Polish experience of implementing a medical examination programme for asbestos-exposed workers and provide a list of activities to consider when planning for such a programme.

## Résumé

En Pologne, l'utilisation de l'amiante a été interdite en 1997 et depuis, les usines d'amiante ont été fermées. Malgré leur fermeture, des cas de maladies professionnelles liées à l'amiante chez les anciens travailleurs de l'amiante continuent d'être enregistrés dans le Registre central des maladies professionnelles. Entre 2001 et 2014, 2726 maladies liées à l'amiante ont été classifiées et consignées dans la catégorie des maladies associées à une exposition professionnelle à l'amiante. En 2000, la Pologne a mis en œuvre un programme appelé *Amiantus* qui vise les anciens travailleurs des usines de transformation de l'amiante. Ce programme a permis aux travailleurs de passer des examens médicaux périodiques et d'avoir gratuitement accès aux médicaments pour le traitement des maladies liées à l'amiante. L'adoption de ce programme a entraîné la collecte de nouvelles données permettant de fournir une estimation fiable du nombre de maladies professionnelles liées à l'amiante, dont le cancer. La période de latence moyenne pour l'asbestose, le cancer du poumon et le mésothéliome étant d'environ 40 ans, des conséquences sur la santé des anciens travailleurs, imposant un suivi, risquent encore de se manifester. Nous présentons l'expérience menée par la Pologne pour mettre en œuvre un programme d'examens médicaux destiné aux travailleurs exposés à l'amiante et fournissons une liste d'activités à prendre en compte au moment de concevoir ce type de programme.

## Resumen

En Polonia, el uso del amianto se prohibió en 1997 y, desde entonces, las plantas de tratamiento del amianto están cerradas. A pesar de su cierre, siguen registrándose casos de enfermedades laborales relacionadas con el amianto entre los antiguos trabajadores de este material. Entre 2001 y 2014, se detectaron 2 726 casos de enfermedades relacionadas con el amianto, clasificadas y registradas como enfermedades asociadas a la exposición laboral al amianto en el registro central de enfermedades laborales. En el año 2000, Polonia presentó un programa titulado *Amiantus*, dirigido a antiguos trabajadores de plantas de tratamiento del amianto. El programa ofreció pruebas médicas periódicas a los trabajadores y acceso gratuito a medicamentos para el tratamiento de enfermedades relacionadas con el amianto. La introducción del programa ofreció información adicional para generar una estimación fiable del número de enfermedades laborales relacionadas con el amianto, incluido el cáncer. El periodo de latencia media de la asbestosis, cáncer de pulmón y mesotelioma es de unos 40 años, por lo que aún puede existir cierto impacto para la salud de los antiguos trabajadores y, por tanto, necesitan someterse a un seguimiento. Se presenta la experiencia polaca acerca de la implementación de un programa de pruebas médicas para trabajadores expuestos al amianto y se ofrece una lista de actividades a considerar a la hora de planificar dicho programa.

## ملخص

تم حظر الأسبستوس في بولندا في عام 1997 كما خضعت منشآت تصنيع المنتجات من الأسبستوس إلى الإغلاق منذ ذلك الحين. وبالرغم من إغلاق تلك المنشآت، ما زال يشهد السجل المركزي للأمراض المهنية تسجيلات لحالات من الإصابة بأمراض مهنية تتعلق بالأسبستوس لدى العاملين الذين سبق لهم العمل في تصنيع المنتجات من الأسبستوس. وظهرت في الفترة بين عامي 2001 و2014 أمراض تتعلق بالأسبستوس بلغ عددها 2726، حيث تم تصنيفها والإبلاغ بها كأمراض ترتبط بالتعرض إلى الأسبستوس بسبب طبيعة المهنة. وطرحت بولندا في عام 2000 برنامجًا باسم *"أميانتوس" استهدف العاملين الذين سبق لهم العمل بمنشآت تصنيع الأسبستوس، * حيث وفّر هذا البرنامج للعاملين الفحص الطبي الدوري والحصول على الأدوية مجانًا لعلاج الأمراض المتعلقة بالأسبستوس. ووفر طرح ذلك البرنامج معلومات إضافية تساعد على ظهور تقديرات موثوق بها لعدد الأمراض المهنية المرتبطة بالأسبستوس، والتي تشمل مرض السرطان. وتبلغ مرحلة الكمون في داء الأسبست، وسرطان الرئة، وورم المتوسطة ما يقرب من 40 عامًا، لذا قد لا تزال هناك بعض الآثار السلبية على الصحة تهدد العاملين السابقين، مما يستوجب خضوعهم للمتابعة الطبية. وإننا نطرح الخبرة البولندية في مجال تطبيق برنامج الفحص الطبي على العاملين ممن سبق لهم التعرض للأسبستوس، ونوفر قائمة بالأنشطة التي يجب النظر فيها في حال التخطيط لمثل هذا البرنامج.

## 摘要

波兰于 1997 年禁止使用石棉，并随之关闭了石棉厂。 尽管关闭了石棉厂，但是根据职业病登记中心的记载，前石棉厂工人中仍有人出现石棉相关职业病。 在 2001 到 2014 年间，共有 2726 例石棉相关疾病被归类为和列为职业性接触石棉相关疾病。 2000 年，波兰针对前石棉加工厂工人推出了一项名为*石绒 (Amiantus)* 的计划。 该计划为工人提供定期体检，并且免费提供治疗石棉相关疾病的药物。 该计划的推出为准确估计石棉相关职业病（包括癌症）患者的人数提供了补充数据。 石棉肺、肺癌和间皮瘤的平均潜伏期大约为 40 年，因此前工人可能仍然存在某些健康隐患，有必要进行后续监测。 我们介绍了波兰计划中为石棉接触工人实施体检计划的具体内容，并且列举了规划此类项目时需要考虑的活动。

## Резюме

В Польше применение асбеста было запрещено в 1997 году, и с тех пор предприятия, работающие с асбестом, закрыты. Несмотря на прекращение их деятельности, в Центральном реестре профессиональных заболеваний по-прежнему регистрируются случаи заболеваний, связанных с асбестом, среди бывших работников предприятий асбестовой промышленности. За период между 2001 и 2014 годами 2726 случаев болезней, связанных с асбестом, были классифицированы и зарегистрированы как болезни, связанные с воздействием асбеста на рабочем месте. В 2000 году Польша предложила программу под названием Amiantus, предназначенную для бывших работников асбестообрабатывающих предприятий. Для работников программа предусматривала периодическое медицинское наблюдение и свободный доступ к лекарственным препаратам для лечения болезней, связанных с асбестом. В результате внедрения программы были получены дополнительные данные, с помощью которых была дана достоверная оценка заболеваемости профессиональными болезнями, связанными с асбестом, в том числе раком. В среднем латентный период асбестоза, рака легких и мезотелиомы составляет приблизительно 40 лет, поэтому негативное воздействие на здоровье бывших работников по-прежнему не исключено и им требуется последующее врачебное наблюдение. Авторы описывают опыт Польши в плане реализации программы медицинского наблюдения для работников, подвергавшихся воздействию асбеста, и перечисляют мероприятия, которые необходимо учесть при разработке такой программы.

## Introduction

Poland banned the use of crocidolite asbestos in the mid-eighties and in 1997 introduced a parliamentary Act banning the use, import and production of asbestos and asbestos-containing products.^1^ Despite the ban, delayed adverse health effects of past exposure from asbestos use and at asbestos-containing material-production sites, continue to be an issue. Some of the health effects include lung cancers and mesothelioma. These biological effects of asbestos on health – which may manifest many years after occupational exposure – necessitate that former asbestos plant workers undergo a medical examination and regular monitoring of related illnesses.

Due to the long latency period – that is the period of time between the first exposure to asbestos and a disease diagnosis – the health effects of occupational exposure to asbestos dust remain subjects of research interest.^2^ In previous studies, the reported mean latency period for asbestos-related diseases, including mesothelioma, is about 30 years.^3^^–^^5^

Because of its carcinogenic nature and pneumoconiosis-generating properties, asbestos dust is considered one of the most dangerous types of dust for workers’ health. The World Health Organization (WHO) estimates that globally about 125 million people are exposed to asbestos in their workplace and each year more than 107 000 deaths are attributable to occupational exposure to asbestos.^6^

WHO and the International Labour Organization (ILO) have taken joint action to improve workers’ health and oblige the Member States of the European Region of WHO to develop national programmes for elimination of asbestos-related diseases by 2015.^7^^,^^8^ The joint action by WHO and the ILO is an important milestone in the implementation of the 2010 Parma Declaration on Environment and Health adopted at the Fifth Ministerial Conference on Environment and Health.^9^ The declaration specifies that a national programme for elimination of asbestos-related diseases should include: a policy; a national asbestos profile; directions for awareness raising and capacity building an institutional framework; and a national plan of action for elimination. A national asbestos profile would include the prevalence of asbestosis and the incidence of lung cancer and mesothelioma among exposed workers.^10^ The European Parliament resolution of 14 March 2013 on “asbestos-related occupational health threats and prospects for abolishing all existing asbestos” obliges its Member States to establish a formal, systematic registration of all asbestos-related diseases. Registration systems for such diseases in many European countries are either poor or non-existent.^11^

Evidence from ecological studies shows that national asbestos consumption, reported as average per capita asbestos use, predicts the incidence of asbestos-related diseases in different populations.^12^ However, comparing asbestos use and asbestos-related disease burden between countries is difficult due to differences in national reporting of such diseases. For example, a 2014 study on asbestos use and asbestos-related diseases in the WHO European Region reported that countries that have banned asbestos reported more asbestos-related deaths, compared to those that have not.^12^ The difference in reporting is most likely due to misdiagnosis and underreporting of asbestos-related diseases in the latter group of countries.^12^ In addition, insufficient knowledge about asbestos-related diseases, lack of information on the negative health effects of past exposure to asbestos, lack of motivation and fear of the consequences of reporting by former workers, as well as lack of specialized knowledge among treating physicians, may constitute reasons for underreporting in countries that have not banned asbestos use. Most cases of mesothelioma are found in low- and middle-income countries that use asbestos-containing products, which are also the same countries where affected populations are less likely to have access to diagnostic tests and regular health care.^13^ In this paper, we present the Polish experience in implementing a prophylactic medical examination programme for asbestos-exposed workers.

## Level of asbestos exposure

In Poland, until the mid-seventies, various tasks in most asbestos processing plants were performed manually. The highest concentrations of asbestos dust and fibre were found at textile plants, slightly lower concentrations in insulation product plants and the lowest in asbestos-cement plants and friction productsplants.^14^ Based on archival data from asbestos-processing plants, it is estimated that over 43 600 individuals have been occupationally exposed to asbestos dust.^14^ It is also estimated that after World War II, 2 million tonnes of asbestos were used by manufacturing plants that produce asbestos-containing products, of which 90% were chrysotile and about 10% crocidolite.^14^ The amount of imported asbestos, which represents annual consumption, was about 1.7 kg per capita, during the period of the highest consumption to date.^14^ This ranks Poland among countries of low-level asbestos consumption.

Eight large state-owned plants processed about 82% of the total asbestos imported to Poland.^14^ Production of asbestos-cement was the largest activity for the sector and the main material used for its manufacturing was chrysotile. Until the mid-eighties, considerable quantities of crocidolite and small amounts of amosite were used to produce pressure pipes.^15^ The highest concentrations of asbestos dust were reported in preparation departments, at workstations to bury material, crushing wheels and where products are cut and polished.

## Definition, diagnosis and reporting

Polish regulations specify the procedure for diagnosis and medical certification of an occupational disease and provide a list of the relevant occupational diseases covered.^16^ According to these regulations, a disease is classified as an occupational disease if it has been caused by a health hazard(s), is present in the work environment and if it is included in the list of occupational diseases. The list of asbestos-related occupational diseases includes: asbestosis; diseases of the pleura or pericardium induced by asbestos dust (diffuse thickening of the pleura, diffuse plaques of the pleura or pericardium, pleural exudate); chronic obstructive pulmonary disease; malignant neoplasms (lung cancer or bronchus cancer, and pleural and peritoneal mesothelioma). 

Asbestosis is the main occupational disease diagnosed among Polish workers exposed to asbestos dust.^17^ In Poland, asbestosis is diagnosed in one of two ways: (i) based on X-ray changes in the lungs according to the International Labour Organization 1980 classification system,^18^ ILO category 1/1 and existing pleural abnormalities or a higher category; or (ii) based on radiological and clinical criteria (radiological findings ILO category 1/1 and existence of at least one other clinical manifestation, for example, crackles at the base of the lungs, restrictive or mixed ventilatory impairment, reduction of oxygen pressure, reduced diffusion capacity or reduction in the static lung compliance). The procedure of certification of an occupational disease case comprises three stages: (i) reporting the suspected case; (ii) assigning a diagnosis to the case; and (iii) certifying the case as an occupational disease. Depending on the workplace location, the employer, an occupational physician or a labour inspector is required to report the case to a local sanitary inspector. Once the certification is completed, the local sanitary inspector documents each occupational disease case on a special form and refers them all to the Central Register of Occupational Diseases located at the Nofer Institute of Occupational Medicine in Lodz. The register provides the government with a comprehensive national database of asbestos-related occupational diseases. Systematic collection of the data on asbestos-related diseases is important for well-informed occupational policies, prevention and compensation.^19^^,^^20^

## *Amiantus* programme

In 2000, the Polish Ministry of Health introduced the *Amiantus* programme of prophylactic medical examination for former workers of 28 asbestos-processing plants. The passing of the Act on the ban on use of asbestos-containing products from 1997 was the basis to start the programme.^1^ Under the programme, former workers of the Polish asbestos-processing plants specified in the Act are entitled to periodic medical examination and free access to medications for treatment of asbestos-related illnesses. The raw material used at an asbestos processing plant where a former employee worked is the criterion to get into the programme. Because of lack of accurate information on the status of periodic exposure to asbestos, industries such as construction and shipyards were not included in the programme. In addition, the *Amiantus* programme provides additional data for measuring the incidence of asbestos-related diseases and for epidemiological research on the long-term effects of occupational exposure to asbestos. The funds to cover the costs of the programme come from the state budget.

Thirteen regional occupational medicine units are implementing the programme. All units are required to perform clinical, radiometric, spirometric and histologic examination according to the 1997 Helsinki criteria for diagnosing asbestos-related diseases.^21^ Once a year, employees undergo a medical examination made up of: a general medical examination; chest X-ray imaging; and resting spirometry and supplementary testing such as resting gasometry and computed tomography scan. The Nofer Institute of Occupational Medicine coordinates and supervises the *Amiantus* programme. Data from the programme are recorded in the Reference Center for Asbestos Exposure and Health Risk Assessment. More information about the *Amiantus* medical monitoring programme and the associated data collection system is available elsewhere.^17^

## Reported cases

According to data from the Central Register of Occupational Diseases, the average latency period for asbestosis, lung cancer and mesothelioma is about 40 years. This indicates a need for long-term follow-up of occupationally-exposed workers. In Poland, despite the former asbestos workers’ prior asbestosis diagnosis or smoking history, each case of lung cancer with a documented exposure to asbestos is compensated as an occupational disease.

Between 2001 and 2014, each year, about 1700 former employees took part in Central Register medical examinations by the *Amianitus* programme, of whom approximately 10% were examined for the first time. The programme has increased the number of reported cases to the Central Register of Occupational Diseases and the detection of pathologies associated with asbestos exposure. However, it is estimated that during the 14 years of the programme’s duration, only about 20% of eligible former asbestos workers were examined.

During the same period, there were 2726 asbestos-related illnesses classified and reported in the Central Register of Occupational Diseases as diseases associated with occupational exposure to asbestos. The most prevalent diseases were: asbestosis (53.3%; 1453), lung cancer (14.6%; 398) and pleural or peritoneal mesothelioma (10.6%; 289). Diseases of the pleura or pericardium were introduced into the list of occupational diseases in 2002 and accounted for 20.5% (559) of cases. 

When comparing data from the register for the period 2001 to 2010 with data from before the *Amiantus* programme (1991–2000), the number of recorded asbestos-related diseases increased almost twofold and in the case of mesothelioma, almost threefold during the implementation of the programme (Table 1). The high reported number of cases for the period between 2001 and 2010 could be because of two factors: (i) the long latency period of the reported diseases; and (ii) increased surveillance by the *Amiantus* programme, which increased their detectability. The high number of reported cases coded as “other non-malignant diseases” and “other neoplasms” could be explained by changes to the relevant list of occupational diseases, which has occurred over time (Table 1). Before the introduction of the programme in 2001, asbestos processing plant workers constituted less than 70% of the diagnosed asbestosis cases and by 2014, they constituted 80%. The programme has contributed to improving the level of awareness about the consequences of asbestos dust exposure and reporting from asbestos processing plants, as well as raising the competency of physicians diagnosing asbestos-related diseases. In addition, providing education to workers that participate in the programme about the risk of tobacco use and the importance of smoking cessation may result in reducing the risk of respiratory diseases in this group over time.

**Table 1:** Average number of certified asbestos-related occupational disease cases per year, Poland, 1971–2014

Years	Type of occupational disease						
	Asbestosis	Lung cancer	Mesothelioma^a^	Diseases of pleura or pericardium^b^	Other neoplasms	Other non-malignant diseases^c^	Total
1971–1980	13.8	0.4	0.2	Not recorded	0.1	0	14.5
1981–1990	77.1	5.4	1.5	Not recorded	2.9	1.3	88.2
1991–2000	70.2	17.3	6.8	Not recorded	8.2	5.4	107.9
2001–2010	122.2	30.6	19.5	42.7	1.7	1.2	217.9
2011–2014	57.7	23.0	23.2	32.7	0	0	136.6

^a^ Five cases are peritoneal mesothelioma.

^b^ Listed as an occupational disease since 2002 and recorded by the Central Register of Occupational Diseases since 2003.

^c^ Includes nine cases of chronic obstructive bronchitis and three cases of chronic atrophic, hypertrophic rhinitis and allergic rhinitis, pharynx, larynx and trachea, which are induced by strong irritant and sensitizing substances, and since 2002 are no longer on the list of occupational diseases.

Data source: Nofer Institute of Occupational Medicine, Lodz, Poland.

Between 2001 and 2013, work-related mesotheliomas constituted about 9% (260/2808) of the nationally diagnosed mesothelioma cases. As seen in other countries, the incidence of occupational mesothelioma can be underestimated due to difficulties associated with diagnostics and linking the disease to workplace exposure.^22^^–^^24^ Lack of in-depth interviews with concerned patients about their occupational history constitutes the main reason for the low incidence of mesotheliomas in former asbestos workers.^17^Follow-up of all asbestos-exposed workers in the country, even after they stopped working, is needed to ensure complete reporting of asbestos-related diseases. Based on the experience gained through the *Amiantus* programme, we provide a list of activities to consider when planning for such a programme in Box 1.

Box 1 **List of activities to consider when planning a medical examination programme for asbestos- exposed workers**

**Policy formulation**

- Stakeholder dialogue.
- Intersectoral collaboration between relevant ministries, such as health and labour.
- Implementing public awareness campaigns.
- Providing information to employees of companies where asbestos-containing material production has been terminated.
- Providing information to affected employees about their entitlement to prophylactic medical examination.

**Legislation**

- Introduction of legislation focused on minimizing exposure to hazardous material and allowing early retirement for asbestos-exposed workers.
- Creation of a national compensation fund and procedures which recognize work-related asbestos cases as an occupational disease.
- Making the documentation of employment history a legal obligation before the closure of an asbestos-containing product-producing plant.
- Making documentation of additional information mandatory, including: the profile of the asbestos-containing product-producing plants; a list of people who have ever been employed at the plants; asbestos exposure assessment history of the plants; production, type and usage of asbestos in the plants.
- Developing and implementing a prophylactic examination programme for former workers of closed plants.

**Coordination**

- Establishing national registers of workers exposed to asbestos.
- Developing a national asbestos-related diseases register.
- Establishing a coordination centre. Tasks may include standardization of medical records and creating a database of key information for epidemiological analysis.

**Monitoring**

- Defining asbestos-related pathology under study, for example, having a criterion for diagnosis of asbestos-related diseases and assessment of asbestos exposure.
- Periodically reviewing and updating the list of asbestos-related diseases under surveillance.
- Developing a questionnaire for a comprehensive medical examination which includes radiological criteria to determine early diagnosis of radiological changes caused by asbestos exposure.
- Harmonizing medical protocols used to examine affected workers.

**Training**

- Introducing the list of asbestos-related diseases to medical practitioners.
- Upgrading physicians’ skills in chest X-ray reading.

**Research**

- Epidemiological research on the long-term effects of occupational exposure to asbestos.
- A cohort study among asbestos-exposed workers to determine health effects and to assess death risk.

## Conclusion

Despite asbestos plant closures, cases of asbestos-related occupational diseases among former asbestos workers are still recorded in Poland. Introduction of the *Amiantus* programme allowed the Polish Ministry of Health to provide former asbestos-plant workers with periodic medical examinations and free access to medications for treatment of asbestos-related illnesses. Introduction of the programme also allowed the government to generate a reliable estimate of the number of asbestos-related occupational diseases, including cancer. Having national legislation that bans asbestos use and mandates access to medical examination for former workers who are exposed to harmful working conditions resulted in the identification of people with known occupational exposure to asbestos and with a developed asbestos-related disease. The detailed exposure information obtained during the examination process has contributed to improved diagnosis of asbestos-related diseases. Lengthening the period of medical observation has allowed identification of asbestos-related diseases among individuals who are of retirement age. A follow-up of all asbestos-exposed workers in the country, even after they have stopped working, is needed to ensure complete reporting of asbestos-related diseases.
